# Laparoscopic distal gastrectomy for gastric cancer in a patient with situs inversus: a case report

**DOI:** 10.1186/s40792-022-01550-2

**Published:** 2022-10-07

**Authors:** Kei Sato, Junki Yamada, Naohito Meguro, Hiroshi Onishi, Kanechika Den, Hiroyuki Baba, Mitsutaka Sugita

**Affiliations:** Department of Surgery, Yokohama City Minato Red Cross Hospital, 3-12-1 Shinyamashita, Naka-ku, Yokohama, Kanagawa Prefecture 231-8682 Japan

**Keywords:** Situs inversus, Gastric cancer, Laparoscopic gastrectomy

## Abstract

**Background:**

Situs inversus (SI) is a rare congenital condition characterized by organ transposition from their normal positions. Careful preoperative planning is important for the safe operation of patients with SI because only a few surgeons have operated on such patients. Here, we report the case of a patient with SI who underwent laparoscopic distal gastrectomy (LDG) with D2 lymph node dissection (LND) for advanced gastric cancer (GC).

**Case presentation:**

The patient was a 72-year-old man diagnosed with GC. Upper endoscopy revealed a type 3 tumor in the anterior wall of the stomach body. Multidetector computed tomography showed no obvious GC metastasis or inverted organs. The preoperative diagnosis was cStage IIB (i.e., cT3, cN0, and cM0) GC with SI. Although liver retracting and intracorporeal suturing required special attention, LDG with D2 LND and Billroth-I reconstruction were safely performed by reversing the usual procedure. The patient was discharged 10 days after the surgery.

**Conclusions:**

To safely perform laparoscopic surgery for GC in patients with SI, sufficient preoperative preparation is necessary. In particular, a reversible method of liver retraction should be prepared.

## Background

Situs inversus (SI) is a rare congenital condition in which the major visceral organs are horizontally reversed from the normal position; however, its etiology remains unclear. The incidence of SI is estimated to be 0.005─0.02% of the general population (1, 2). SI is classified into situs inversus totalis (SIT), which refers to the total transposition of the thoracic and abdominal organs, and situs inversus partialis (SIP) in which the organs are partially mirrored. Among the 2 types of SI, SIT accounts for about 90%, and SIP is extremely rare (3). Several surgeons have reported performing gastrectomy for gastric cancer (GC) in patients with SIT (4, 5). However, there has only been one report of gastrectomy for GC in a patient with SIP (6). It was a report of robot-assisted distal gastrectomy for GC in a patient with SIP (6). We describe the case of laparoscopic distal gastrectomy (LDG) for GC in a patient with SIP and discuss important recommendations for a safe operation.

## Case presentation

**Fig. 1:**
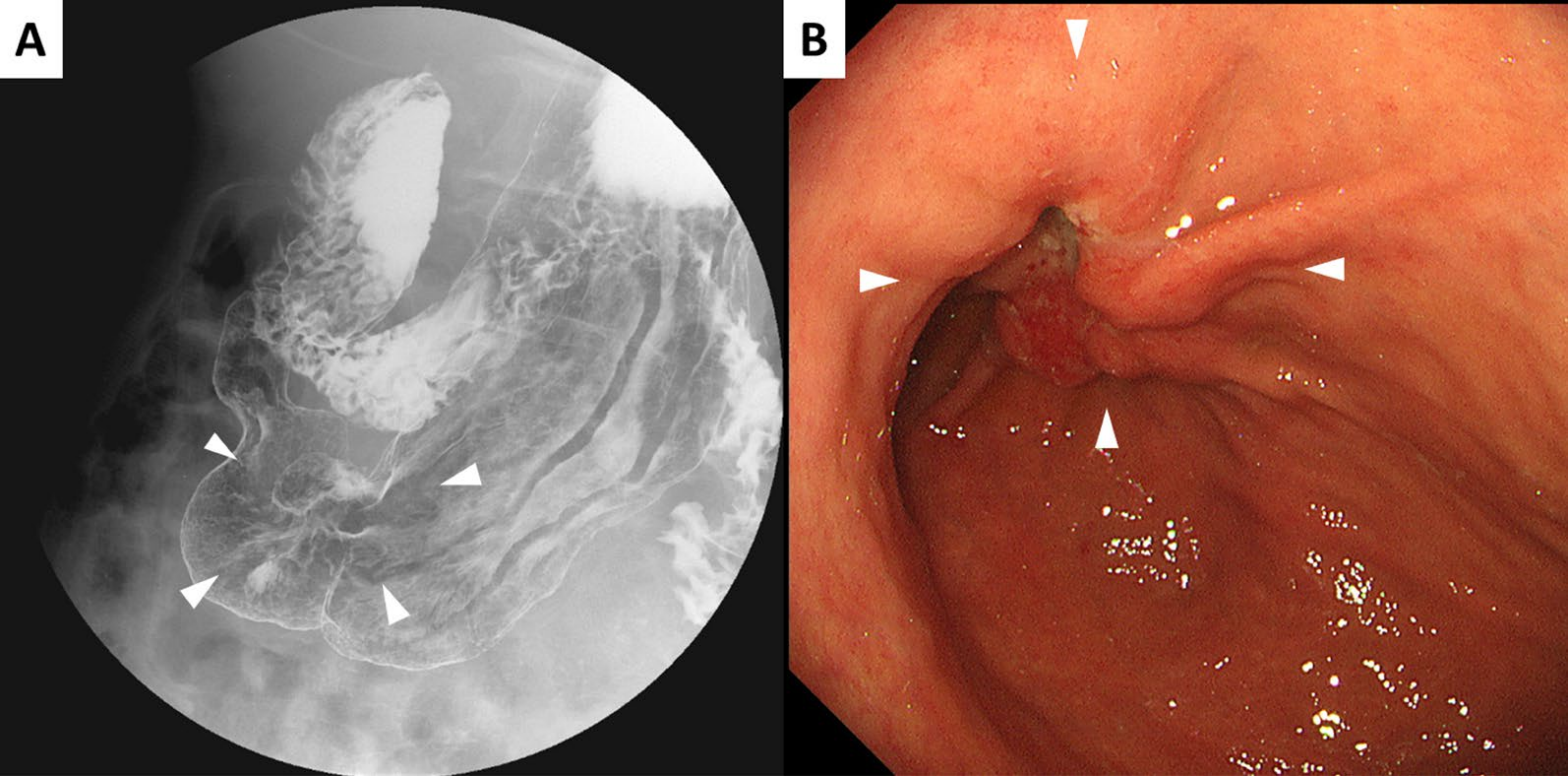
**A** Upper gastrointestinal imaging of the double contrast study in the prone position revealed an inverted image of the upper gastrointestinal tract and an irregular attachment of barium in the anterior wall of the stomach (white arrowhead). **B** Upper endoscopy showed an elevated lesion in the anterior wall of the stomach body (white arrowhead)

**Fig. 2:**
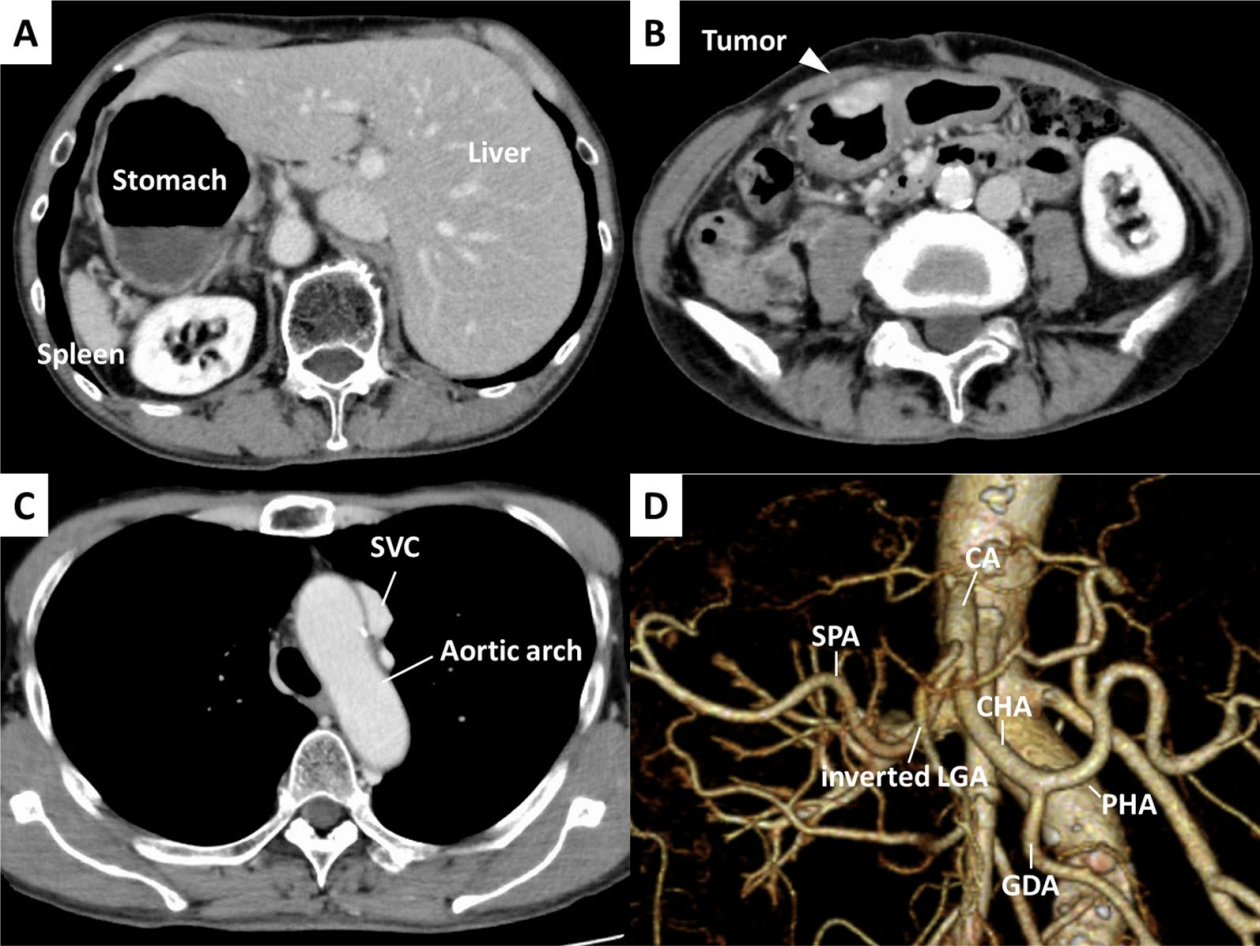
**A** Contrast-enhanced MDCT revealed inverted abdominal organs **B** and illustrated the wall thickness of the stomach body (white arrowhead), which had no obvious metastasis. **C** The aortic arch to the thoracic aorta was in the normal position. **D** The 3D angiography showed a complete right–left reversal of the abdominal arteries. *SVC* superior vena cava, *CA* celiac artery, *CHA* common hepatic artery, *PHA* proper hepatic artery, *GDA* gastroduodenal artery, *SPA* splenic artery, *LGA* left gastric artery

The patient was a 72-year-old man with no pertinent medical history. An upper gastrointestinal imaging (UGI) performed for GC screening (Fig. 1A) revealed an abnormality wherein there was a horizontal inversion of the upper gastrointestinal tract and deformation of the stomach body. Upper endoscopy showed an elevated lesion with an ill-defined border in the anterior wall of the lower-third of the stomach body (Fig. 1B), and histopathological examination revealed a moderately differentiated tubular adenocarcinoma. Contrast-enhanced multidetector computed tomography (MDCT) showed inverted thoracic and abdominal organs and illustrated the wall thickness of the stomach body, which had no obvious metastasis (Fig. 2A, B). Furthermore, the aortic arch to the thoracic aorta was in the normal anatomical position (Fig. 2C); hence, the case was diagnosed as SIP. The 3D angiography revealed a complete right–left reversal of the abdominal arteries (Fig. 2D). SI is known to be frequently associated with chronic sinusitis and bronchiectasis, and this clinical triad is called the Kartagener syndrome (7). However, chronic sinusitis and bronchiectasis were not seen in this patient.

Preoperative staging of GC was clinical Stage IIB (cT3N0M0) according to the tumor/node/metastasis (TNM) classification of malignant tumors 8^th^ edition.

**Fig. 3:**
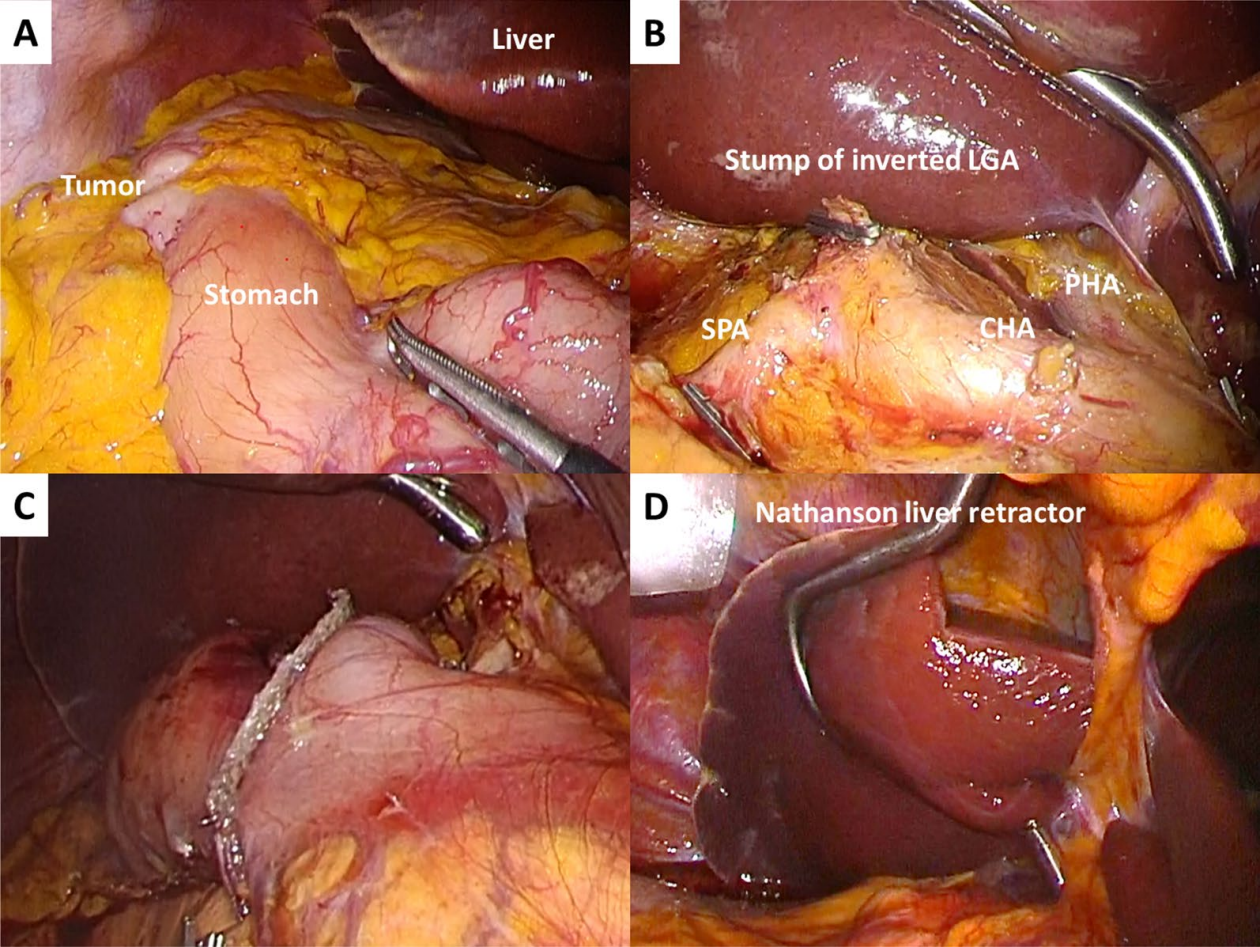
**A** Abdominal organs were completely left–right inverted. **B** Intraoperative findings after D2 LND. **C** Intraoperative findings after modified delta-shaped Billroth-I reconstruction. **D** Findings of retracting the lateral segment of the liver with the Nathanson liver retractor

LDG with D2 lymph node dissection (LND) and modified delta-shaped Billroth-I reconstruction was performed. Although this case was SIP, the abdominal organs were completely inverted (Fig. 3A); hence, this operation could be performed with the usual LDG symmetrically (Fig. 3B, C). Five trocars were placed in the left–right reversal of the usual LDG in our hospital. The surgeon and the assistant performed this operation by reversing the standing position and the roles of the left and right hands from the usual LDG. The surgeon had to operate the energy device with the non-dominant hand. However, the surgeon used the energy device with the dominant hand for supra-pancreatic LND. The problem encountered with this method was that the forceps of the left hand crossed the energy device; however, the supra-pancreatic LND could be safely performed without the energy device interfering with the pancreas.

In this operation, there were two problems that could not be solved by performing the operation symmetrically. One of the problems was the method of retracting the lateral segment of the liver. We usually use the Nathanson liver retractor in laparoscopic gastrectomy (LG), which was also used in this case. However, the tip of the liver retractor was oriented toward the hepatic hilar side due to its asymmetrical hook shape. Therefore, the retraction of the lateral segment of the liver was somewhat insufficient, and special care was required not to damage the liver (Fig. 3D). The second problem was intracorporeal suturing. We usually perform intracorporeal suturing with the surgeon in the paraxial position; however, in this operation, the surgeon performed the suturing in the co-axial position between the patient’s legs. By shifting to this standing position, the surgeon was able to suture with the dominant hand and the normal needle direction.

Operation time, including intraoperative frozen section histological analysis for the proximal resection margin, was 323 min, and blood loss was 10 ml. After an uneventful postoperative course, the patient was discharged on postoperative day 10. Postoperative staging of GC was pathological Stage IIIB (pT4aN3aM0) according to the TNM classification of malignant tumors 8th edition. The patient received S − 1 + docetaxel adjuvant chemotherapy for 1 year after surgery without any signs of recurrence.

## Discussion

SI is a rare congenital anomaly in which the organs are transposed from their normal anatomical position to the opposite side along the sagittal plane. Careful preoperative planning is important for the safe operation of patients with SI because very few surgeons have operated on such patients.

In recent years, LG has been accepted as the standard treatment not only for early-stage GC but also for advanced GC. Therefore, the procedure for LG in each institution has also been standardized. To be succinct, the LG for GC with SI can be performed by reversing the usual procedure. However, for that purpose, there are some challenges to overcome, such as accurately recognizing the anatomy in the left–right inverted field of view and operating by exchanging the left and right hands. Laparoscopic surgery is usually more difficult than open surgery because of the limitation in the field of view and the angle of the forceps. However, this operation has revealed some advantages of laparoscopic surgery in a patient with SI. The surgeon and the assistants were accustomed to the anatomy of SI by watching the image reproduced by the left–right inversion of the usual LDG prior to the operation. In laparoscopic surgery, it was easy to confirm the anatomy pre- and intraoperatively even in a patient with SI because the surgeon and the assistants can see the same field of view. Laparoscopic surgery also had an advantage in terms of surgical technique. While operating on a patient with SI, the surgeon must use the energy device with the non-dominant hand because the roles of the left and right hands are reversed. However, in laparoscopic surgery, it was possible to operate with more stability even with the non-dominant hand by using the trocar as a fulcrum for operating the energy device.

We have described the surgical procedure for GC with SI by reversing the standing position and the left and right hands from the usual LG. However, some surgeons took a different approach from our method. Namikawa et al. (8) reviewed 16 cases in which laparoscopic or robotic gastrectomy was performed for GC with SIT (5, 8–21). We reviewed their report and added seven cases. The additional cases were as follows: one case (6) with SIP, five cases (4, 22–25) with SIT reported based on their report, and the patient in this study (Table 1). In 5 of these 23 cases, the surgeon performed LG in the same position as usual. In these cases, it appears that the surgeon emphasized the operation with the dominant hand rather than performing the surgical procedure as usual. The details of the intraoperative procedure were unknown; however, no intraoperative or postoperative complications occurred in these five cases. However, as mentioned earlier, we were able to use an energy device even if the patient’s non-dominant hand was used in this study. Therefore, we reasoned that performing the surgery by fully simulating the surgery that reverses the usual LG before the surgery would be safer. Recently, robotic-assisted gastrectomy (RAG) for a patient with SI has been frequently reported. RAG has several advantages for patients with SI. The first advantage of RAG is that the surgeon does not need to change positions, even when operating on a patient with SI, because the surgeon operates on the console. The second advantage of RAG is that there is less need to switch the roles of the dominant and non-dominant hands because robotic instruments have a wide range of motion. This advantage is more pronounced when performing intracorporeal suturing. Takeno et al. (24) performed proximal RAG using the double-flap technique on a patient with SI. It would be extremely difficult to perform double-flap reconstruction on a patient with SI using laparoscopic surgery. However, presently, RAG is still under development and cannot be performed in all hospitals. Therefore, LG remains a useful option for patients with GC and SI.

**Table 1:** Characteristics of gastric cancer patients with situs inversus who underwent laparoscopic or robotic gastrectomy

Case	Author	Year	Age	Gender	Tumor location	Tumor size (mm)	Stage	Vessel anomaly	Position of surgeon	Type of gastrectomy	Lymph node dissection	Liver retraction method	Reconstruction	Operative time (min)	Blood loss (ml)	Postoperative complication
1	Yamaguchi et al.	2003	76	Male	ND	ND	ND	ND	ND	Distal	ND	ND	ND	ND	ND	ND
2	Futawatari et al.	2010	53	Male	L	50	IA	None	Opposite	Distal	D1 +	Snake	Billroth-I	300	350	None
3	Seo et al.	2011	60	Male	L	15	IB	None	Same	Distal	D2	Fan	Billroth-I	200	70	None
4	Kim et al.	2012	47	Male	M	40	IIIB	None	Same	Distal	D1 +	ND	Billroth-I	300	ND	None
5	Fujikawa et al.	2013	60	Female	M	40	IB	None	Opposite	Distal	D1 +	Snake	Billroth-I	234	5	Bowel obstruction
6	Min et al.	2013	52	Male	L	33	IB	CHA from SMA	Same	Distal	D1 +	ND	Billroth-I	220	100	None
7			68	Male	L	32	IA	None	Same	Distal	D1 +	ND	Billroth-I	117	50	None
8	Sumi et al.	2014	42	Male	L	ND	IB	LHA from SMA	Opposite	Distal	D1 +	ND	Billroth-I	313	90	None
9	Ye et al.	2015	60	Male	L	ND	IIB	None	Opposite	Distal	D2	ND	Billroth-I	230	50	None
10	Morimoto et al.	2015	58	Male	U	25	IA	None	Opposite	Total	D1 +	Nathanson	Roux-en-Y	359	90	None
11	Shibata et al.	2016	79	Male	U	80	IIB	RGEA above RGEV	Same	Total	D2	ND	Roux-en-Y	232	100	None
12	Kigasawa et al.	2017	40	Male	L	24	IA	None	Opposite	Distal	D1 +	ND	Billroth-I	284	40	None
13	Alhossaini et al.	2017	52	Female	L	ND	IA	None	Robotic	Distal	D1 +	ND	Billroth-I	195	30	None
14	Dai et al.	2018	53	Male	L	30	IIIB	None	Robotic	Distal	D2	ND	Billroth-I	180	50	None
15	Aisu et al.	2018	64	Female	M	15	IA	CHA and RGEA from 1st jejunal artery	Robotic	Distal	D1 +	Nathanson	Billroth-I	451	150	Hepatopathy and pancreatic fistula
16	Ojima et al.	2019	80	Female	L	20	IB	None	Robotic	Distal	D2	ND	Billroth-I	260	20	None
17	Namikawa et al.	2020	74	Male	M	21	IB	CHA from SMA	Opposite	Distal	ND	Nathanson	Roux-en-Y	335	20	None
18	Yoshimoto et al.	2020	84	Male	U	ND	IIIA	None	Robotic	Total	D2	Internal organ	Roux-en-Y	ND	30	None
19	Abbey et al.	2021	69	Male	L	25	IIIB	None	Robotic	Distal	ND	ND	Roux-en-Y	205	20	None
20	Takeno et al.	2021	71	Female	U	20	IA	None	Robotic	Proximal	D1 +	Internal organ	Double -flap	448	45	None
21	Harada et al.	2021	63	Female	U	ND	IB	None	Opposite	Total	D2	ND	Roux-en-Y	422	30	None
22	Doden et al.	2022	74	Male	L	25	IA	None	Opposite	Distal	ND	ND	Billroth-I	220	100	None
23	The present case	2022	72	Male	L	60	IIIB	None	Opposite	Distal	D2	Nathanson	Billroth-I	323	10	None

*U* upper-third of the stomach, *M* middle-third of the stomach, *L* lower-third of the stomach, *CHA* common hepatic artery, *SMA* superior mesenteric artery, *ND* not described

In this operation, there was a problem with retracting the lateral segment of the liver by the Nathanson liver retractor. The retraction of the lateral segment was somewhat inadequate, and special attention was required not to damage the liver because the retractor has an asymmetrical hook shape. Aisu et al. (6) reported that the Nathanson liver retractor compressed the central portal vein and caused liver ischemia during the robot-assisted distal gastrectomy for the patient with SIP. They also stated that planar liver retraction by the Penrose drain method (26) or disk suspension method (27) may be useful in preventing liver ischemia in SI patients. In addition, Hiramatsu et al. (28) reported the importance of reducing both the localized pressure and liver retraction time when using the Nathanson retractor to prevent postoperative transient liver dysfunction in LG. Our operation did not cause intraoperative liver ischemia or postoperative liver dysfunction; however, we lacked preoperative recognition of the asymmetry and intraoperative attention to localized pressure for liver of the Nathanson retractor. The present case, unlike the case of Aisu et al. (6), had no left-sided gall bladder and the portal vein was arranged abnormally. Therefore, it is likely that our case did not experience intraoperative liver ischemia and postoperative hepatic dysfunction. However, it may have been safer to prepare for another method of liver retraction, such as the disk suspension method.

## Conclusions

To safely perform laparoscopic surgery for GC in patients with SI, sufficient preoperative preparation is necessary. In particular, a reversible method of liver retraction should be prepared.

## Data Availability

Not applicable.

## Abbreviations

GC: Gastric cancer
LDG: Laparoscopic distal gastrectomy
LG: Laparoscopic gastrectomy
LND: Lymph node dissection
MDCT: Multidetector computed tomography
SI: Situs inversus
SIT: Situs inversus totalis
SIP: Situs inversus partialis
UGI: Upper gastrointestinal imaging
RAG: Robotic-assisted gastrectomy
